# Ecology and distribution of the recently discovered ked *Lipoptena andaluciensis* (Diptera: Hippoboscidae) in Spain

**DOI:** 10.1038/s41598-025-26742-2

**Published:** 2025-11-28

**Authors:** Mikel Alexander González, Shirin Taheri, Néstor Martínez-Calabuig, Paloma Prieto, Jordi Figuerola

**Affiliations:** 1 Estación Biológica de Doñana (EBD, CSIC), Sevilla, Spain; 2 Ciber de Epidemiología y Salud Pública (CIBERESP), Madrid, Spain; 3 INVESAGA Group, Department of Animal Pathology, Faculty of Veterinary, University of Santiago de Compostela, Lugo, Spain; 4 Consejería de Sostenibilidad y Medio Ambiente, Junta de Andalucía, Jaén, Spain

**Keywords:** *Lipoptena andaluciensis*, Host prevalence, Ensemble modeling, Species occurrence, Wildlife parasitology, Spain, Ecology, Ecology, Zoology

## Abstract

Hippoboscid flies (Diptera: Hippoboscidae) are obligate hematophagous ectoparasites of birds and mammals, with recognized ecological and epidemiological importance. Despite their relevance, the ecology and distribution of many species within this family remain poorly understood. We combined active screening of 372 wild ungulates (2013–2024) and passive CO₂-baited trap sampling (2023 – 2024) across Andalusia, Spain, to investigate the occurrence, host prevalence, and phenology of *Lipoptena andaluciensis* flies. We used these data to model its potential distribution and spatial overlap with key host species using an ensemble species distribution modeling approach. The presence of *L. andaluciensis* was confirmed in four Andalusian provinces, with the first record dating to 2018. Host prevalence increased significantly in recent years, reaching 69.7% in 2023 and 72.9% in 2024. Roe deer (*Capreolus capreolus*) and fallow deer (*Dama dama*) showed the highest infestation prevalence (70.0% and 50.4%, respectively), followed by red deer (*Cervus elaphus*, 27.2%) and mouflon (*Ovis aries musimon*, 3.9%). No flies were found on Iberian ibex (*Capra pyrenaica*) or wild boar (*Sus scrofa*). Winged adults were active, at least, from April to November, while wingless forms were present on hosts at least from June to March. Species distribution models identified extensive areas of high environmental suitability across central and southern Spain, driven primarily by vegetation density (NDVI), soil moisture, and land surface temperature. The models revealed a strong spatial overlap with the distribution of roe deer and fallow deer. Our findings document the widespread presence and high recent prevalence of *L. andaluciensis* in southern Spain and identify cervids, particularly roe deer, as its primary hosts. The extensive suitable habitat predicted beyond its current known range and the recording of its presence in Italy suggest that the potential distribution is larger than expected and the species may have been overlooked in Spain and probably in other countries of southern Europe. This study provides a critical baseline for monitoring the population dynamics and assessing the ecological and veterinary impact of this neglected ectoparasite.

**Supplementary Information:**

The online version contains supplementary material available at 10.1038/s41598-025-26742-2.

## Background

The geographic distribution of a parasite is fundamentally constrained by the distribution of its potential hosts. Consequently, understanding host-parasite interactions is essential for characterizing parasite’s potential range and forecasting the impacts of environmental change on its distribution^1^.

Among such parasites, flies of the genus *Lipoptena* (family Hippoboscidae), commonly referred to as “deer keds” or “louse flies” are of particular relevance. These ectoparasites are primarily associated with ungulates, including cervids and other wild ruminants, but may also infest domestic livestock and occasionally feed on humans^2,3^. Their veterinary importance is rooted in their ability to cause irritation, skin lesions, and potential secondary infections in hosts, thereby affecting animal health and productivity^4^. Additionally, *Lipoptena* species have been associated with several pathogens of veterinary and zoonotic concern, including *Bartonella* spp., *Anaplasma phagocytophilum*, *Borrelia* spp., *Rickettsia* spp.*, Babesia* spp., *Theileria* spp., *Coxiella burnetii*, *Francisella tularensis, Mycoplasma* spp., and *Trypanosoma* spp, among others^5^.

In 2023, a new species, *Lipoptena andaluciensis,* was discovered in southwest Spain^6^. Specimens initially identified, within the natural area of Sierra de Cazorla, Segura y Las Villas Natural Park, as the exotic species *Lipoptena fortisetosa,* were later reclassified as *L. andaluciensis*^7,8^. Due to its recent discovery and increasing detections across southern Spain, there is an urgent need to understand its basic ecology. To address this knowledge gap, we investigated the seasonal occurrence and temporal changes in the incidence of *L. andaluciensis*. We combined field data with ecological niche models to estimate its potential geographic range and assess the spatial overlap with its putative ungulate hosts across Spain. Species distribution models are critical for newly discovered species like *L. andaluciensis*, where biological information is sparse^9,10^. Such modeling not only delineates current distribution patterns but also identifies areas at risk of future colonization, thereby guiding targeted surveillance and management efforts.

## Material and methods

### Collection of field samples

**Fig. 1:**
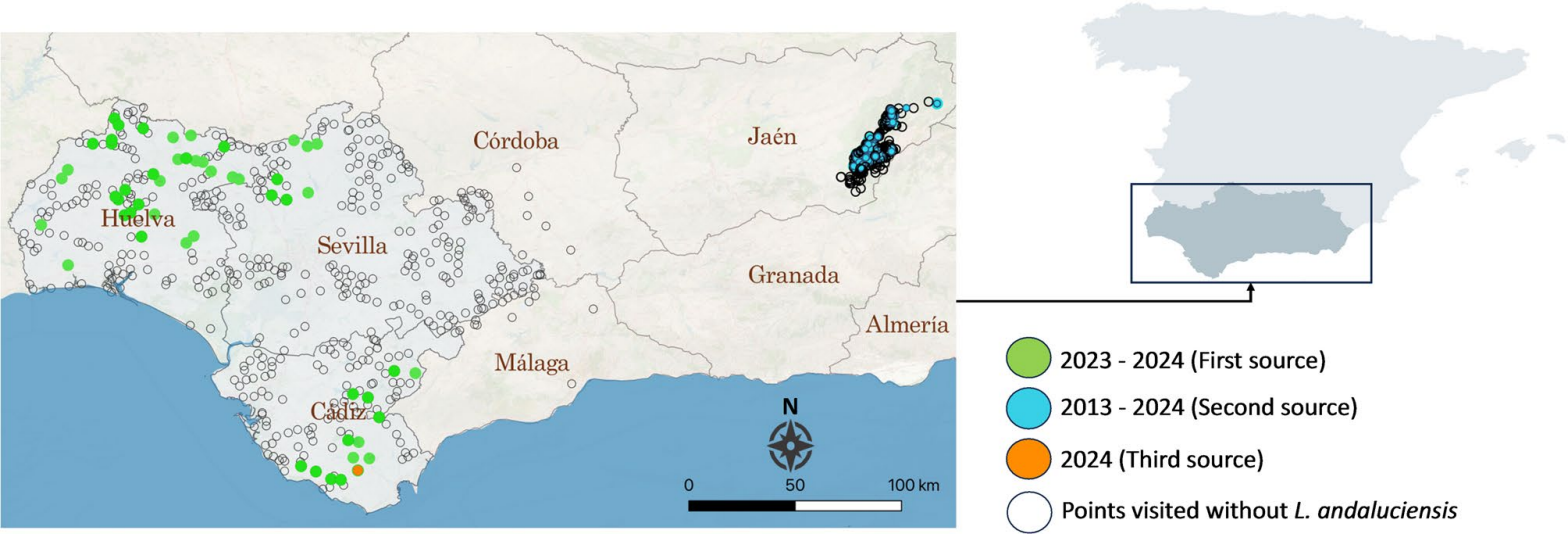
Geographic distribution of sampling sites of *L. andaluciensis* flies in southern Spain, Andalusia (2013 – 2024), classified according to three data sources. Colored dots (green, blue, and orange) show presence of *L. andaluciensis* and uncolored dots indicate absence. For the third data source, there are three absence points located almost in the same position as the orange one. The base map is provided from Streat map License: CC BY-SA 3.0

The material was collected from three different sources within the Autonomous Community of Andalusia in southern Spain (Fig. 1). Andalusia has a Mediterranean climate with hot, dry summers and mild, wet winters. Summer temperatures often exceed 30 °C, while winters range from 8 to 16 °C. Rainfall is highest from October to April, varying from over 2,000 mm in mountainous areas to less than 300 mm in the more arid eastern regions.

The first source of data was the collection of winged adult keds using BG-Sentinel 2 traps (BIOGENTS, Germany) baited with approximately 1 kg of dry ice as a source of CO₂, operating over 24-h periods between April − 2023 and November − 2024, following the same sampling design and trapping protocols described by González et al.^6^. Briefly, the sampling was stratified by habitat using a 5 × 5 km grid covering the provinces of Sevilla, Huelva, and Cádiz, an area spanning approximately 31,614 km^2^. Over the two years, the study included 865 sampling points (440 in 2023 and 425 in 2024). Each sampling point was sampled three times between April and November giving a total of approximately 2.600 trapping days of sampling effort, supplemented by 27 additional sites in the provinces of Málaga and Córdoba, resulting in 892 sampling locations.

The second source of data involved active screening of hunted animals (but also stealth, roadkill, injured specimens, or animals directly trapped alive for routine inspection) across ten municipalities in the province of Jaén within and around the Sierra de Cazorla, Segura y Las Villas Natural Park (Fig. 1). This park, the largest protected area in Spain and a UNESCO Biosphere Reserve, is characterized by rugged mountains, dense forests, and diverse ecosystems. A veterinary team examined individuals from five mammal species between 2013 and 2024. The sample size varied among months depending on the number of specimens available for examination.

The last source of data was derived from the collection of keds from 10 recently hunted 2 – 8 years old male roe deers in July 2024 in Alcornocales Natural Park in Zanona (Los Barrios, Cádiz) (Fig. 1). The habitat is characterized by the predominance of evergreen cork oak (*Quercus suber)* forests and shrub vegetation, particularly *Cytisus scoparius*.

From the second and third sources, examinations were carried out either in the field or in the laboratory to detect the presence of hippoboscids on animals. Keds were manually removed from their hosts using forceps, and a subsample of 5 – 10 specimens per host was preserved in ethanol. All the hippoboscids collected from these three different sampling sources were counted, identified, and sexed according to the recent taxonomic keys for the identification of European hippoboscid species^6,11^.

### Analysis of seasonal change in *L. andaluciensis* prevalence

The presence/absence data collected from the second source of information and the sampling of wild mammals was analyzed with a nominal regression model including year as a continuous independent variable and host species as independent factor (*Capra pyrenaica* Iberian ibex, *Cervus elaphus* Red deer, *Dama dama* Fallow deer, *Ovis aries musimon* Mouflon, and *Sus scrofa* Wild boar).

### Selection of environmental variables

A time series of monthly climate data from the Terraclimate datasets^12^ was used, providing climate information on minimum and maximum temperatures, and precipitation, with a spatial resolution of approximately 4 km. We also considered wind speed, runoff, actual and potential evapotranspiration, soil moisture, vapor pressure, and PDSI (Palmer Drought Severity Index). Variables were averaged over the two years (2023 – 2024) using the R package “dismo”^13^. Land cover variables were obtained from the Copernicus Global Land Operations (CGLOPS-1) dataset. This component of the Copernicus Earth observation program provides global land cover maps with a 100-m resolution, updated annually since 2015. These maps categorize land cover into 23 classes, based on the United Nations Food and Agriculture Organization’s Land Cover Classification System^14,15^. To characterize the environmental composition of species ranges, we calculated the proportion of each land-cover category within 2 × 2 km grid cells across the study area. Land cover was classified into the following main categories: water, trees, flooded vegetation, grassland, cropland, shrub and scrub, built-up areas, bare ground, and snow/ice. All spatial analyses were conducted in RStudio version 4.4.0.

Three key remotely sensed environmental indices derived from MODIS products were integrated: the Normalized Difference Vegetation Index (NDVI), representing vegetation greenness and density; the Normalized Difference Water Index (NDWI), indicative of surface water presence and moisture levels; and Land Surface Temperature (LST), which reflects surface thermal conditions. These indices were obtained for the years 2023 and 2024, corresponding to the biological data collection period. Elevation data were also incorporated using the Copernicus Digital Elevation Model (DEM).

Because the environmental variables originated from different sources with varying original resolutions, we resampled them to a common grid using Inverse Distance Weighting (IDW) interpolation. IDW is a deterministic geostatistical approach that estimates values at unsampled locations by averaging surrounding observations, assigning greater influence to nearby points^15^.

We implemented a structured variable selection procedure to identify a parsimonious set of predictors. First, all environmental variables were screened for multicollinearity. Pairwise correlations were examined, and for highly correlated pairs (|r|> 0.8), the variable with greater ecological relevance or stronger univariate association with *L. andaluciensis* occurrence was retained. We then calculated Variance Inflation Factors (VIF) for the remaining variables and iteratively removed those with VIF > 10 until all retained variables had VIF < 5. This procedure follows established recommendations for minimizing multicollinearity^16^.

From this reduced pool, we constructed biologically plausible candidate models with different combinations of variables, fitted within the *sdm* package in R^17^. Model performance was evaluated using repeated cross-validation and bootstrapping (200 iterations each). To assess predictive performance, we used metrics appropriate for presence–background data, specifically the True Skill Statistic (TSS) and the Area Under the Receiver Operating Characteristic Curve (AUC). Variables that consistently contributed more strongly to model performance (measured through the contribution scores within the SDM framework) were preferentially retained^18,19^.

Guided by the principle of parsimony, we selected the final set of variables (Table S1) by balancing predictive performance (TSS) with ecological interpretability, retaining only those predictors that contributed meaningfully to the models^20,21^. Therefore, models with higher (TSS) were selected as final models.

After applying variable selection and conducting multicollinearity tests, the reduced set of variables was used to fit the final model.

### Modelling potential distribution of *L. andaluciensis*

We modelled the potential distribution of *L. andaluciensis* in relation to climate and land-use variables using an ensemble modelling approach implemented through the *sdm* package in R^17^, combining outputs from four widely applied species distribution modeling techniques: Random Forest (RF^22^;), Generalized Additive Models (GAM^23^;), Maximum Entropy (MaxEnt^24^;), and Polynomial Generalized Linear Models (GLM^25^;). This framework integrates models that differ in assumptions and algorithmic structures, improving overall predictive performance and providing robust, ecologically meaningful insights into the species’ potential distribution^26^.

Since independent data for testing were unavailable, we employed two data splitting methods: bootstrap sampling and cross-validation to evaluate model accuracy, with 200 replications for each method. Because the species points are presence-only, we generated background points throughout the entire study area with 10,000 random backgrounds with 10 times repetition with a certain geographical distance from the presence points^27^. Background points were sampled randomly within a 2 km radius of the presence points.

We projected the model to Spain, using environmental data from Andalusia as a training set. We assessed environmental similarity between the training set and the projection area using the Multivariate Environmental Similarity Surface (MESS) index^28^. This analysis identifies areas where environmental conditions fall outside the range of the training data, highlighting regions where model predictions should be interpreted with caution because projecting models over novel environmental conditions can compromise reliability^29^.

To assess model performance and describe the variation across runs, we first report the full range and variability of TSS values, including models with TSS < 0.75, to provide a transparent overview of ensemble performance. For the predictive mapping, however, we applied a more restrictive criterion: only models that achieved a mean TSS ≥ 0.75 were retained for generating the final consensus distribution map. These well-performing models were combined using a weighted averaging approach, with weights proportional to their respective TSS values. We selected TSS as our primary evaluation metric because it simultaneously accounts for sensitivity (true positives) and specificity (true negatives), and is considered more robust than other commonly used indices for evaluating predictive performance in species distribution models.

### Niche overlap of mammalian hosts with *L. andaluciensis*

We analyzed the distribution of the four most common potential host species of *Lipoptena* genus in Spain^6^: *C. elaphus*, *Capreolus capreolus* (Roe deer), *D. dama*, and *O. aries musimon* in relation to various environmental variables.

The mammal occurrence data were obtained from Palomo et al.^30^, which is based on data collected between 1991 and 1999 and provided at a spatial resolution of 10 km. To ensure temporal consistency, we first modeled the species distributions using climate and land-use data from the same time period. To determine the potential distribution of four mammals across Spain, the trained models were projected onto environmental conditions from 2023 to 2024, aligning temporally with the occurrence records of *L. andaluciensis*, using *sdm* package in R^17^. Climate and land-use variables were processed using the same workflow as for the parasite, ensuring consistency across models.

After modelling the potential distributions of *L. andaluciensis* and the four selected mammalian host species, we conducted a spatial niche overlap analysis to identify areas of overlap and ecological similarity. To quantify niche overlap, we used Schoener’s D^31^, a widely used metric that measures the absolute similarity between two species’ probability distributions across environmental gradients implemented in the *sdm* package in R^17^. This value ranges from 0 (no overlap) to 1 (complete overlap), providing a direct estimate of shared environmental preferences.

All analyses in this study were performed in R version 4.4.2^32^ and the maps were produced using QGIS 3.40 (QGIS Development Team, 2024).

## Results

### Current distribution

A total of 119-winged *L. andaluciensis* specimens (71 females and 48 males) were collected between 2023 and 2024 across 21 municipalities in the provinces of Sevilla, Huelva, and Cádiz, at elevations ranging from 5 to 644 m above sea level (masl) (Fig. 1). Additional samples from vertebrate hosts revealed the presence of unwinged *L. andaluciensis* specimens (n = 64 specimens) in one municipality from Cádiz (155 – 511 masl) and 90 specimens collected from five municipalities in Jaén (822 – 1430 masl) (Fig. 1). No other louse species were collected except one *Pseudolynchia canariensis* in the first source of data.

### Infestation prevalences in mammalian hosts

*Lipoptena andaluciensis* parasitized at least four mammal species, including three Cervidae (*C. elaphus, D. dama,* and *C. capreolus*) and one Bovidae subspecies (*O. aries musimon*). A total of 372 individuals from six different mammal species were inspected between 2013 and 2024 (Table 1). Based on this survey, the first record of *L. andaluciensis* occurred in 2018 on a *D. dama* in Jaén (southeast Spain). The overall parasite prevalence was 22.5% (n = 84/372) (Table 1). *Capreolus capreolus* showed the highest infestation prevalences, with 70% (n = 7/10) of the animals sampled being infested. *Dama dama* displayed also higher rates (50.4%, n = 59/117), followed by *C. elaphus* (27.2%, n = 15/55), and *O. aries musimon* (3.9%, n = 3/76). No *L. andaluciensis* specimens were found in *S. scrofa* (n = 0/30) and *C. pyrenaica* (n = 0/84) (Table 1). The prevalences increased over time, with less than 15% from 2013 to 2020 (χ^2^ = 102.08, 1 df, *p* < 0.0001), and higher percentages were recorded over the last two years (2023 = 69.7% and 2024 = 72.9%) (Table 1).

**Table 1:** Summary of wild host species screened for the presence of *L. andaluciensis* from 2013 to 2024 in Jaén and in Cádiz (southern Spain) in 2024.

nº	Host species	Year								
		2013- 2017	2018	2019	2020	2021	2022	2023	2024	Σ T
84	*Capra pyrenaica*	0 (22)	0 (1)	0 (18)	0 (7)	0 (15)	0 (17)	0 (3)	0 (1)	0 (84)
30	*Sus scrofa*	0 (3)	0 (1)	0 (1)	0 (4)	0 (3)	0 (9)	0 (2)	0 (7)	0 (30)
55	*Cervus elaphus*	0 (14)	0 (2)	1 (10)	1 (8)	1 (7)	5 (6)	6 (6)	1 (2)	15 (55)
117	*Dama dama*	0 (10)	1 (1)	1 (13)	2 (17)	10 (16)	4 (19)	15 (16)	26 (25)	59 (117)
76	*Ovis aries musimon*	0 (5)	0 (9)	0 (12)	0 (22)	0 (7)	0 (12)	2 (6)	1 (3)	3 (76)
10	Capreolus capreolus*	0 (0)	0 (0)	0 (0)	0 (0)	0 (0)	0 (0)	0 (0)	7 (10)	7 (10)
nº of parasitized hosts		0	1	2	3	11	9	23	35	84
nº of hosts		54	14	54	58	48	63	33	48	372
% infestation		0	7.1	3.7	5.2	22.9	14.3	69.7	72.9	22.5

Parentheses indicate the total number of hosts analyzed. * Hosts were analyzed in the province of Cádiz only in 2024.

At the beginning of the study period (2018 – 2021), hosts were infected by a low numbers of flies, and infestation prevalences may have been underreported. After 2022, most parasitized animals contained higher numbers of *Lipoptena* flies (dozens of specimens per individual host), and even one *C. elaphus* and one *D. dama*, were infected by hundreds of specimens. Keds were primarily located on the ventral side of the animals, but smaller numbers were found across the entire body beneath the fur.

### Phenology

**Fig. 2:**
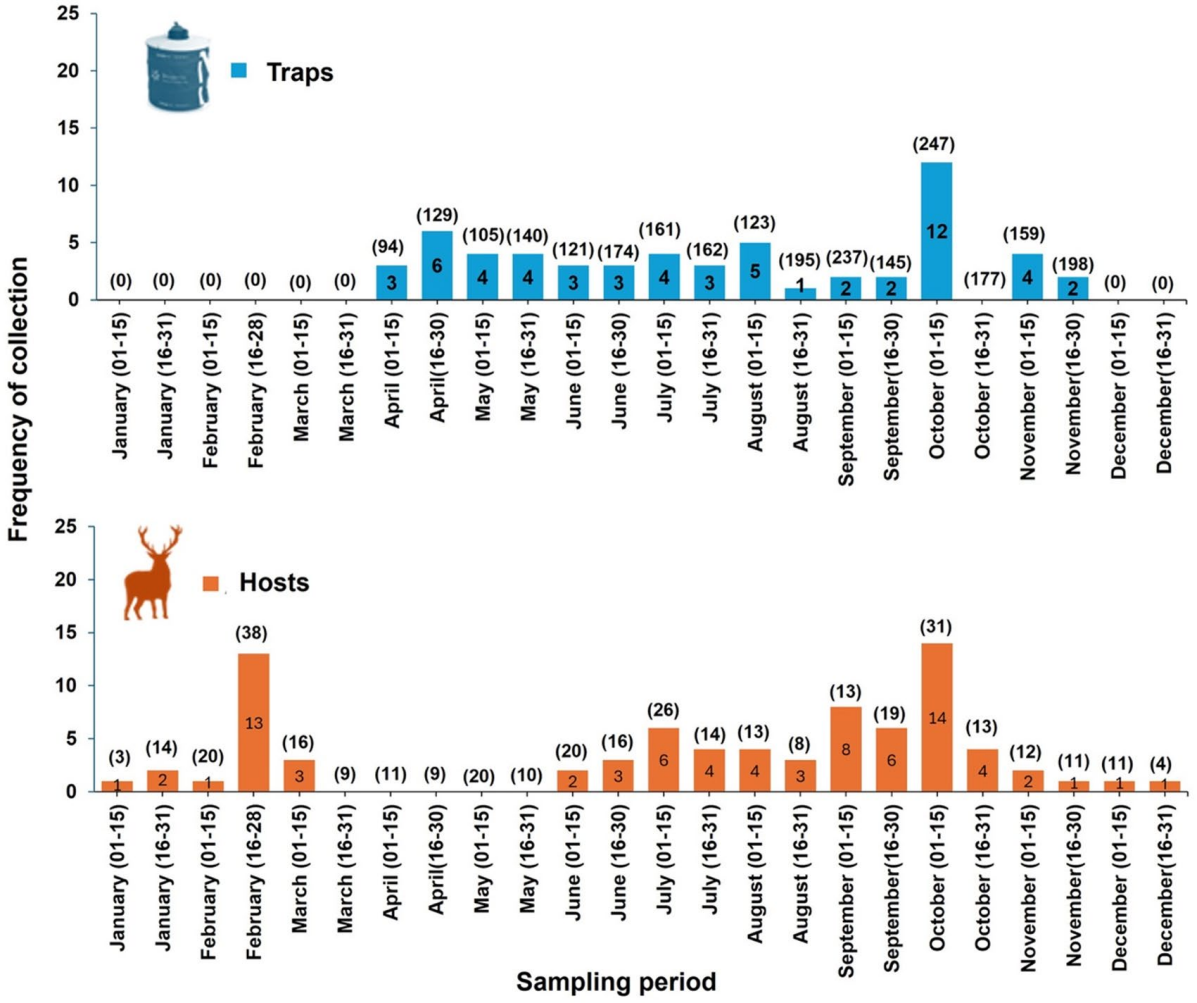
Frequency of collection of *L. andaluciensis* with CO_2_ baited traps between 2023 and 2024 (above) and from three mammal host species (*D. dama*, *C. elaphus,* and *O. aries musimon*) between 2013 and 2024 (below). Infestations in *C. capreolus* were excluded from this graph because all the individuals were captured in July 2024. Parenthesis above the bars represent the monthly sampled sizes for traps and hosts, respectively. Three positive samples collected on hosts were excluded from the analysis because their dates contained errors.

Our data indicates that winged *L. andaluciensis* flies remain active at least from early April to late November, whereas wingless forms were collected throughout the entire year except in April and May (Fig. 2). Based on CO_2_ baited traps, the highest prevalences were found in the month of April (4.0%), May (3.3%), and October (2.8%). Moreover, regarding *L. andaluciensis* found on hosts, the highest monthly infestation prevalence was recorded in September (43.7%) and October (40.9%). *Lipoptena andaluciensis* was not observed on hosts between late March, April, and May, however the number of mammals examined during these periods was low (Fig. 2).

### Modelling *L. andaluciensis* distribution

**Fig. 3:**
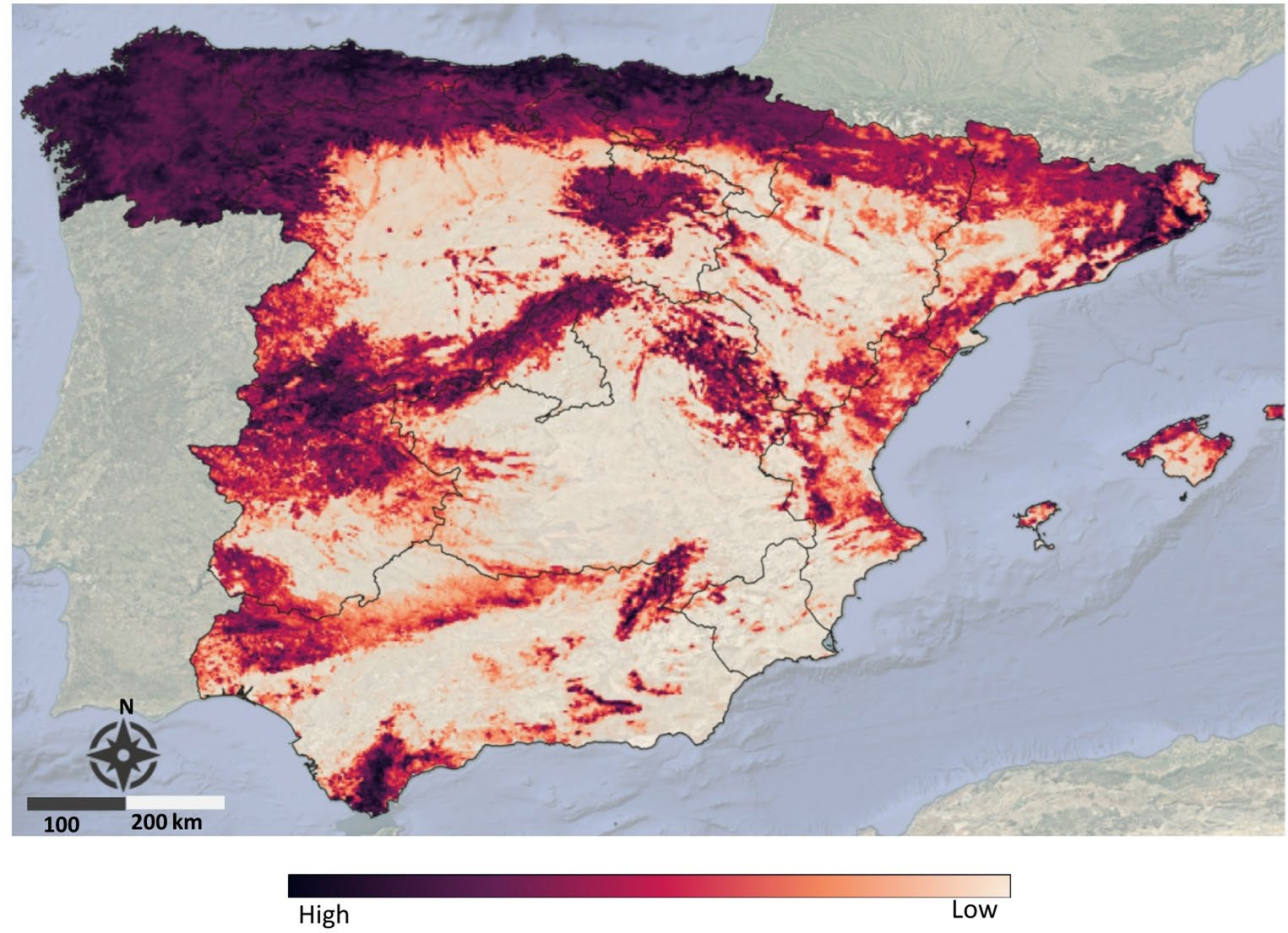
The potential distribution of *L. andaluciensis* across Spain. Darker red areas indicate a higher probability of occurrence and clear colors low or no suitability. The map shows the predicted distribution generated using an ensemble of four modeling techniques implemented in R version 4.4.2^32^. Cartographic visualization was produced using QGIS version 3.40 (QGIS Development Team, 2024).

Ensemble model projections indicate a broader potential distribution of *L. andaluciensis* including extensive areas in the south, central, and north Spain. More suitable areas are concentrated in regions dominated by Iberian coniferous forests and semi-natural landscapes, particularly those with dense herbaceous vegetation, moderate elevation, high soil moisture, and low anthropogenic disturbance (Fig. 3).

**Fig. 4:**
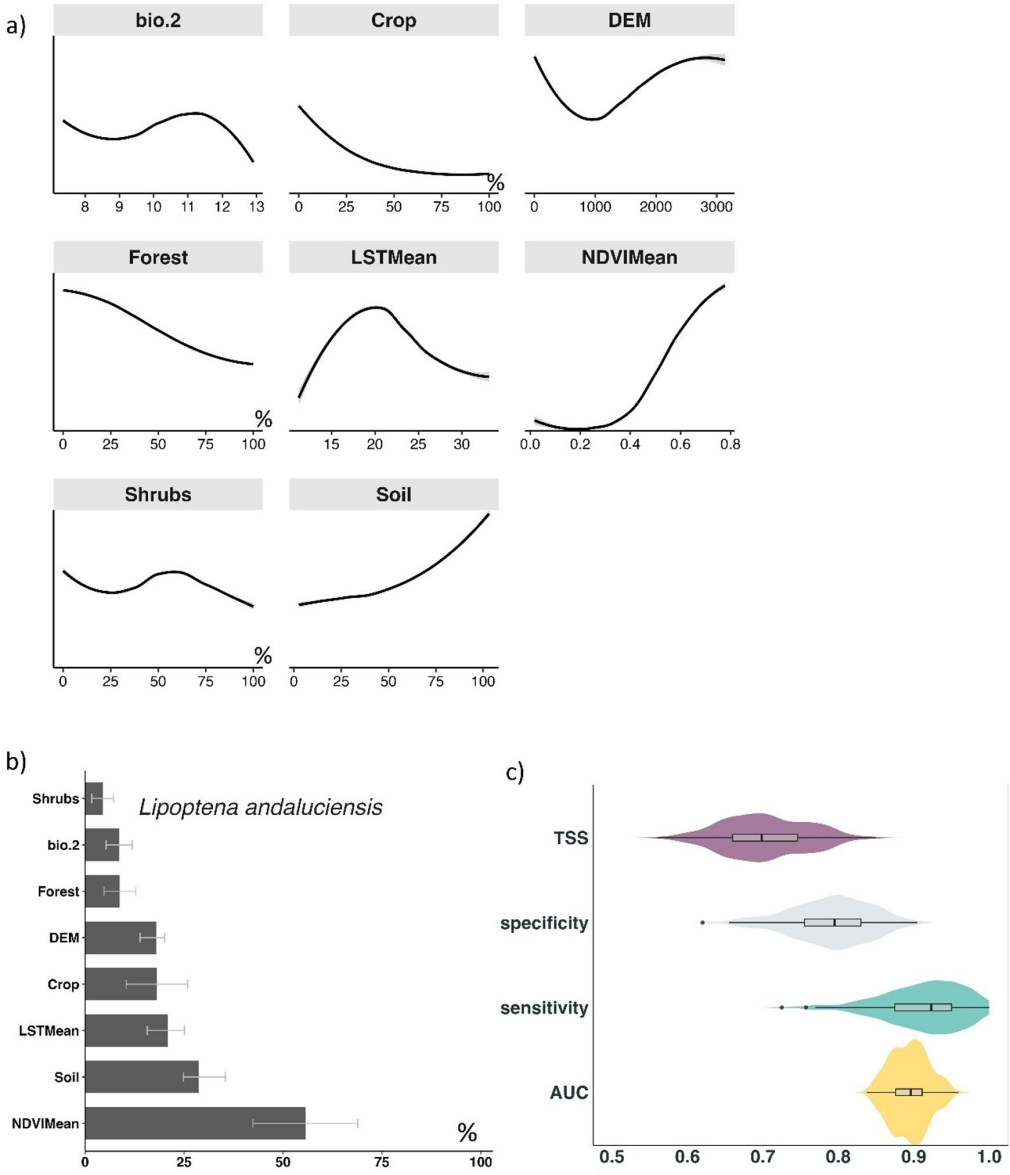
(**a**): Response curves illustrate the relationship between the probability of occurrence of *L. andaluciensis* (Y-axis) and different environmental variables. (**b**): Variable importance (%Inc MSE) indicating the relative contribution of eight environmental variables to the habitat suitability model: NDVI Mean (mean NDVI during 2023 – 2024), Soil (soil moisture), LST Mean (mean land surface temperature during 2023 – 2024), Crop (percentage of crops in 2 km grids), DEM (digital elevation model), Forest (percentage of forest area in each 2 km grid), Bio.2 (mean diurnal temperature range), and Shrubs (percentage of shrub cover in each 2 km grid). **(c)**: Statistical performance of the ensemble model for *L. andaluciensis*, including True Skill Statistic (TSS), Area Under the Curve (AUC), Sensitivity (true positives), and Specificity (true negatives).

The potential distribution of *L. andaluciensis* is strongly positively associated to NDVI, soil moisture, and LST. The results suggested that the species is more closely associated with vegetation density and greenness than with broad land cover categories. In contrast, cropland areas were negatively associated with the species’ presence (Fig. 4a and b).

Overall, the model demonstrated strong predictive performance, with an average AUC of 0.89 and a TSS of 0.74, indicating good accuracy and reliability in predicting the potential distribution of *L. andaluciensis* (Fig. 4c). These metrics reflect strong discrimination between presence and absence locations, fair predictive power, and a good balance between sensitivity (true positives) and specificity (true negatives).

### Modelling the current distribution of potential hosts

**Fig. 5:**
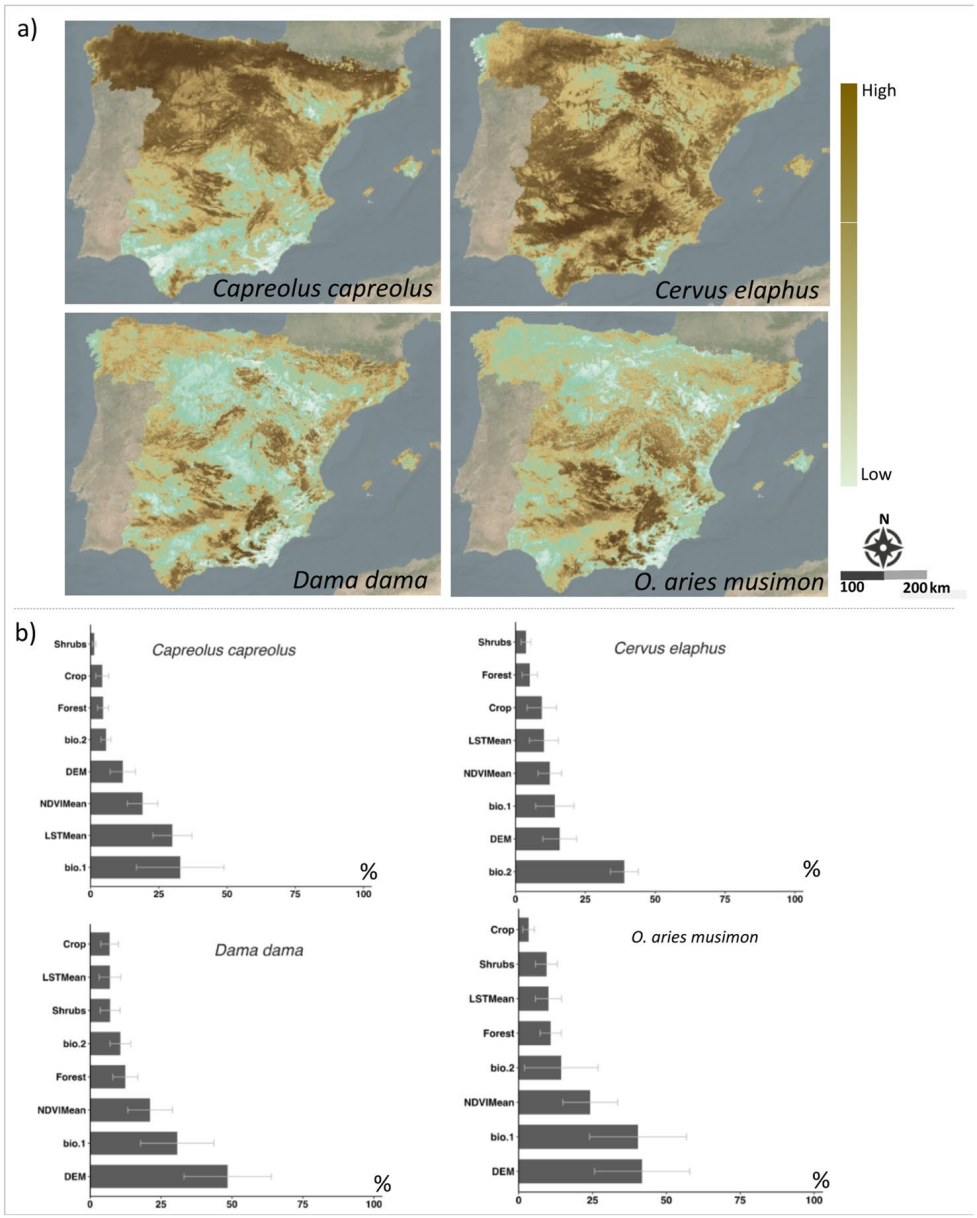
(**a**) Models of the potential distribution of four mammal species host of *L. andaluciensis*. (**b**) Relative importance of environmental variables influencing habitat suitability for each mammal species.

The potential distribution of *C. capreolus, C. elaphus, D. dama* and *O. aries musimon* was modelled (Fig. 5). Overall, the distribution of these species coincides with previous records, although the four mammal species occupy distinct ecological niches, their potential distributions are primarily influenced by similar environmental factors, including annual mean temperature, elevation, forest cover, and NDVI (Fig. 5a and b). See Additional files: Figure S1 and Figure S2 for model performance parameters and how similar the environmental conditions, respectively.

### Spatial overlap between *L. andaluciensis* and its potential hosts

**Fig. 6:**
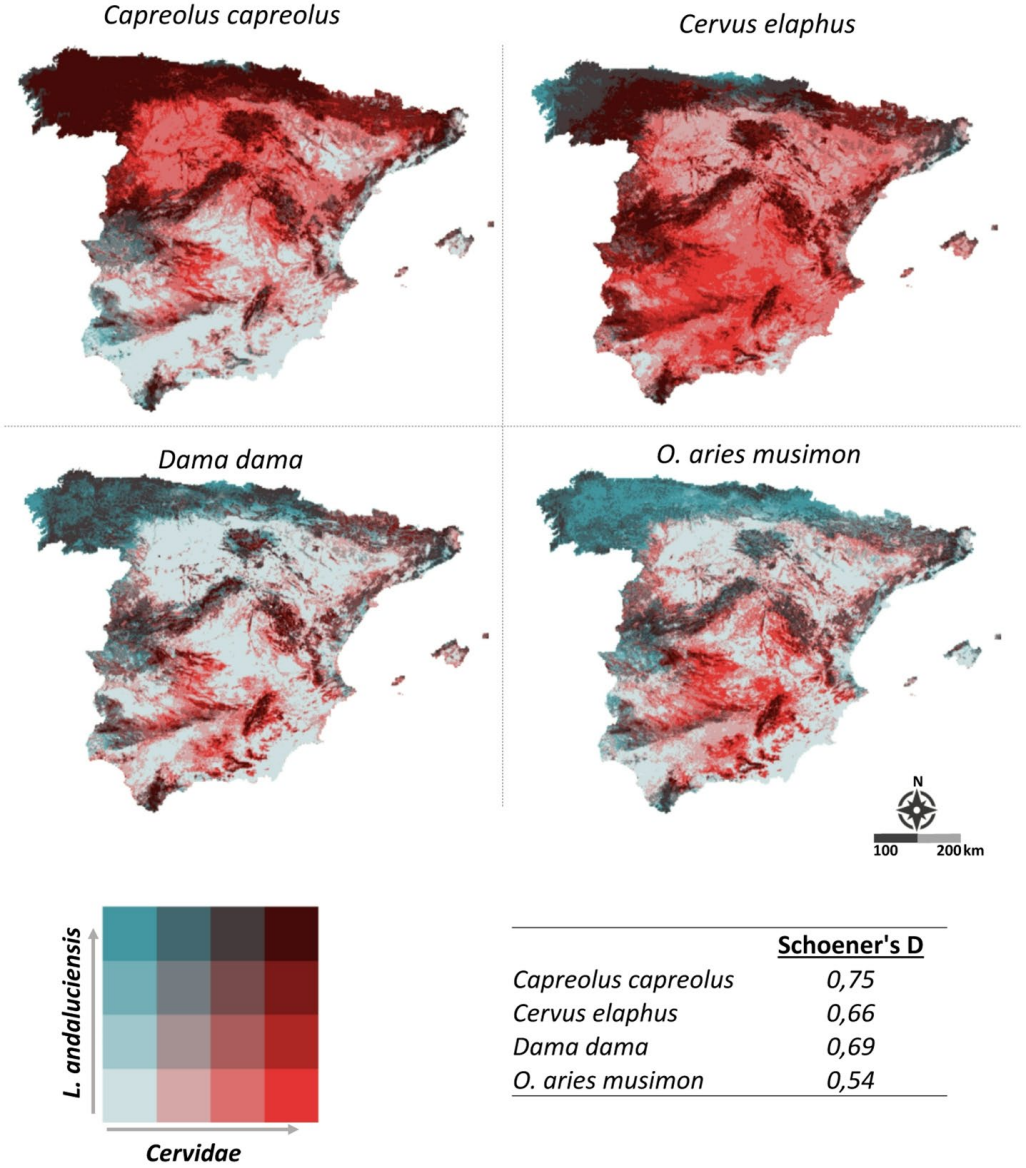
Spatial overlap between *L. andaluciensis* and the four mammalian species. The bivariate choropleth map highlights areas of co-occurrence, with darker shades indicating regions of higher overlap between the parasite and its potential hosts. Schoener’s D values are provided for each mammalian species, with higher values indicating greater spatial overlap with *L. andaluciensis.* The spatial overlap between the four host species and *L. andaluciensis* was assessed and mapped using R version 4.4.2^32^.

Our analysis indicates varying degrees of spatial overlap, ranging from moderate to high (Fig. 6). The highest overlap was observed with *C. capreolus* (D = 0.75), suggesting a strong spatial association and likely suitability as a host. *Dama dama* (D = 0.69) and *Cervus elaphus* (D = 0.66) also showed considerable overlap, indicating potential host-parasite interactions within shared habitats. In contrast, *O. aries* *musimon* exhibited the lowest overlap (D = 0.54), indicating a comparatively weaker spatial association with the ectoparasite (Fig. 6).

## Discussion

Our study offers insights into the infestation dynamics of *Lipoptena* flies across a range of host species. Although we did not specifically analyze parasite abundance, our observations showed clear differences in the parasite prevalence among host species. The highest prevalences were recorded in *C. capreolus* and *D. dama*, and to a lesser extent in *C. elaphus* and *O. aries musimon*. Conversely, no keds were found on *S. scrofa* or *C. pyrenaica*. Because all host species were sampled within a relatively confined geographic area, differences in prevalence are more likely to reflect intrinsic host preferences of *L. andaluciensis*, particularly toward cervids, rather than strong environmental contrasts.

Winged *L.andaluciensis* individuals were observed throughout most of the year, with high activity recorded in spring and autumn. Unfortunately, we lack information on keds from December to March, as BG traps used for the capture of winged adults were only operated from April to November. Therefore, we cannot confirm or rule out adult dormancy during the coolest months. Regarding specimens attached to hosts (unwinged flies), parasitized animals were recorded in all months except during some specific periods, particularly April and May. These observations suggests that *L. andaluciensis* may exhibit prolonged host-seeking activity in southern Spain. Its seasonal cycle might resemble that of tropical or subtropical regions, where multiple generations per year are common rather than the patterns seen in countries with stronger environmental seasonality^33^. In other cooler areas of Europe, the seasonal occurrence of *Lipoptena* species is largely influenced by climatic conditions^34,35^, with active periods typically extending from early spring to late autumn.

Our models suggest that *L. andaluciensis* potential habitat could cover the entire country, including much of northern Spain. This region, characterized by mountainous terrain and dense vegetation, may provide suitable habitats for the species, facilitating its presence. However, MESS map showed that environmental conditions, particularly in northern Spain, represent high environmental dissimilarity, meaning conditions there differ substantially from the model’s calibration data (Additional file: Figure S3). Despite the scarce information available until now, it seems that the species is not yet present in northern areas. It is worth noting that range expansions have been documented in other *Lipoptena* species, such as *Lipoptena cervi* and *L. fortisetosa,* in several parts of Europe due to natural movements or to the translocation of infected hosts^36–39^. In our study, *L. andaluciensis* is predicted to occur in forested habitats or vegetated areas, however the model’s response curve did not show a strong correlation with the forest land cover variable. This suggests that the species distribution might be more dependent on specific vegetative characteristics, as captured by NDVI, than on whether an area is formally classified as forest.

The habitat suitability inferred for *L. andaluciensis* showed the highest overlap with *C. capreolus*, a forest-associated species that also presented a high prevalence of infestation in our samples, supporting its role as a primary host. Overlap with *C. elaphus* and *D. dama* was moderate, while *O. aries musimon* showed the lowest overlap, reflecting the narrower ecological niche of *L. andaluciensis* and its dependence on microclimatic conditions in forested and vegetated landscapes.

Despite ecological differences among the hosts, their distributions were associated with broadly similar climatic and vegetation gradients. Consistently, the environmental niche overlap analysis revealed higher values for environmental than for geographic overlap. Among the host species, the highest overlap was observed with *C. capreolus*, followed by *C. elaphus* and *D. dama*, whereas *O. aries musimon* showed the lowest overlap. These results emphasize the central role of host availability, particularly *C. capreolus* in shaping the ecological niche of *L. andaluciensis*.

The data we present suggest that *L. andaluciensis* is moderately distributed in specific habitats of southern Spain. Based on host collection records, we hypothesize that the prevalence of *L. andaluciensis* has been increasing in recent years, with the earliest known records of this ked in the region dating back to around 2018 in Jaén. This finding raises an important question: does *L. andaluciensis* represent an autochthonous species that remained undescribed until recently and is now increasing in incidence? or alternatively, it is a recent introduction of a non-native species that is still expanding its distribution and abundance in Spain? The distribution of some *Lipoptena* species worldwide has been strongly influenced by the movement of their preferred hosts, primarily cervids, which serve as their main source of blood meal^3,40^. However, the recent detection of the species in Italy in 2023 and 2024, and the retrospective analyses showing its presence in Italy at least since 2007^41^ further supports that the species may present a wider geographical distribution and its presence has remained unnoticed for years, because it was misidentified with other species. At present, we lack sufficient evidence to determine if *L. andaluciensis* is an autochthonous species in Europe that has remained unnoticed until now, a previously undescribed African species that has extended its distribution in Europe, see as example^42^, or an alien species that has reached and stablished in Europe from an overseas unknown origin.

Despite the novel insights provided, this study has some limitations that should be acknowledged. First, sampling of vertebrate hosts was strongly influenced by animal availability and hunting restrictions, meaning that certain months are underrepresented. This seasonal gap reduces our ability to fully characterize the annual cycle of *L. andaluciensis*. Second, although both active and passive sampling methods were employed, we lacked independent datasets to externally validate our results. The absence of such independent testing introduces some uncertainty in our findings, particularly regarding the robustness of the species distribution models. Our results should be interpreted with caution in areas where environmental conditions differ substantially from those used to calibrate the models. The MESS analysis revealed high environmental dissimilarity in parts of northern Spain, indicating that these regions fall outside the range of the training data. Predictions in such areas are therefore less reliable, as the models are forced to extrapolate beyond observed conditions. While we present the full projection across Spain for completeness, we emphasize that suitability estimates in environmentally novel regions should be regarded as tentative and subject to further validation with independent occurrence data. However, the presence of *L. andaluciensis* in Italy^41^ strongly supports our conclusion that its distribution is geographically larger than previously assumed.

Another limitation of this study is that the mammal occurrence data used to model host distributions were derived from atlas records collected during the 1990s. Although we addressed temporal mismatches by calibrating models with environmental conditions from the same period and then projecting them onto current (2023 – 2024) climate and land-use layers, we acknowledge that host populations may have shifted over the past three decades. Species such as fallow deer and mouflon, in particular, have experienced demographic changes and potential range expansions in parts of Spain, which could influence present-day overlap with *L. andaluciensis*. While temporal projections provide a widely accepted and practical approach in SDMs, some uncertainty remains, and future studies would benefit from updated, high-resolution host distribution data to refine these predictions.

.

From a management perspective, infestation by *Lipoptena* flies can cause significant harm to its hosts, through irritation, painful bites, skin inflammation, alopecia, and secondary infections^43^. Such infestations represent a growing concern for wildlife management, particularly for cervids that may suffer from long-term health effects. Additionally, the bite of keds may present a potential threat to human health, as it is capable of causing dermatitis, allergic reactions, and in severe cases, anaphylactic shock^44–46^. Although human-biting behavior has not yet been observed in *L. andaluciensis*, we observed winged specimens hovering around veterinarians and field operators, suggesting potential human exposure.

## Conclusions

*Lipoptena andaluciensis* presence is geographically wider than originally described. Models show suitable habitats in most Spain for this species and potential for further spread highlighting the need for better understanding of its origin and its potential impact on animal health. Different ungulates are infected by the species while *C. capreolus* and *D. dama* hosts showed higher prevalences. The prevalence of infestation has also increased during the last years, and additional studies are needed to clarify its role in the transmission of pathogens to wildlife and its direct and indirect impacts on health.

## Supplementary Information

Below is the link to the electronic supplementary material.

Supplementary Material 1

## Data Availability

All data analyzed during this study are included within the paper and the supplementary files.

## Abbreviations

RF: Random forest
GAM: Generalized additive models
GLM: Generalized linear models
PDSI: Palmer drought severity index
VIF: Variance inflation factor
NDVI: Normalized difference vegetation index
LST: Landscape surface temperature
DEM: Copernicus digital elevation model
CGLOPS-1: Copernicus global land operations
TSS: True skill statistic

## References

[1] Simberloff, D. & Krasnov, S. M. B. Oxford University Press, 2010. 288 pp. (ISBN 9780199561353 paper). *Bioscience* **61**, 925–927 (2011).

[2] Reeves, W. K. & Lloyd, J. E. Louse flies, keds, and bat flies (Hippoboscoidea). *Med. Vet. Entomol.* **3**, 421–438. 10.1016/B978-0-12-814043-7.00020-0 (2019).

[3] Dibo, N., Yang, Y., Wu, X. & Meng, F. A brief review on deer keds of the genus *Lipoptena* (Diptera: Hippoboscidae). *Vet. Parasitol.* **313**, 109850. 10.1016/j.vetpar.2022.109850 (2023). 36473321 10.1016/j.vetpar.2022.109850

[4] Andreani, A. Study on Diptera Hippoboscidae of the genus *Lipoptena*, parasites of ungulates, and morphological and bioecological investigations on *L . fortisetosa*, a new species for Italy. Ph. Dissertation. (Universitá Degli Studi Firenze, Italy, 2022).

[5] Bezerra-Santos, M. A. & Otranto, D. Keds, the enigmatic flies and their role as vectors of pathogens. *Acta Trop* **209**, 105521. 10.1016/j.actatropica.2020.105521 (2020). 32447028 10.1016/j.actatropica.2020.105521

[6] González, M. A. et al. Molecular and morphological analysis revealed a new *Lipoptena* species (Diptera: Hippoboscidae) in southern Spain harbouring *Coxiella burnetii* and bacterial endosymbionts. *Vet. Parasitol.* **332**, 110300. 10.1016/j.vetpar.2024.110300 (2024). 39270602 10.1016/j.vetpar.2024.110300

[7] Carles-Tolrá, M., Yerro, P. P. & Baena, M. *Lipoptena fortisetosa* Maa, 1965: especie nueva para la península ibérica (Diptera: Hippoboscidae). *Rev. Gad. Entomol.* **14**, 27–37 (2023).

[8] Carles-tolrá, M., Yerro, P. P. & Baena, M. Sobre la identidad real de *Lipoptena fortisetosa* Maa, 1965 en la península ibérica (Diptera: Hippoboscidae). *Rev. Gad. Entomol.* **16**, 11–13 (2025).

[9] Franklin. J. & Miller, J. A. Mapping species distributions: Spatial inference and prediction. Mapping Species Distributions: Spatial Inference and Prediction. 1–320. 10.1017/CBO9780511810602. (2010).

[10] Peterson, A. T., Papeş, M. & Soberón, J. Mechanistic and Correlative Models of Ecological Niches. *Eur J Ecol* **1**, 28–38. 10.1515/EJE-2015-0014 (2015).

[11] Oboňa, J. et al. Updated taxonomic keys for European Hippoboscidae (Diptera), and expansion in Central Europe of the bird louse fly *Ornithomya comosa* (Austen, 1930) with the first record from Slovakia. *Zookeys* **1115**, 81–101. 10.3897/ZOOKEYS.1115.80146 (2022). 36761073 10.3897/zookeys.1115.80146 PMC9848778

[12] Abatzoglou, J. T., Dobrowski, S. Z., Parks, S. A. & Hegewisch, K. C. TerraClimate, a high-resolution global dataset of monthly climate and climatic water balance from 1958–2015. *Scientific Data.* **5**, 1–12. 10.1038/sdata.2017.191 (2018). 29313841 10.1038/sdata.2017.191 PMC5759372

[13] Hijmans, R. J., Phillips, S., Leathwick, J. & Elith, J. Species distribution modeling [R package dismo version 1.3–16]. CRAN: Contributed packages. 10.32614/CRAN.PACKAGE.DISMO (2024).

[14] Buchhorn M, Smets B, Bertels L, Lesiv M, Tsendbazar N-E, Herold M, et al. Copernicus Global Land Service: Land Cover 100m: collection 2: epoch 2015: Globe n.d. 10.5281/ZENODO.3243509.

[15] Shepard D. A two-dimensional interpolation function for irregularly-spaced data. Proceedings of the 1968 23rd ACM national conference on -, New York, New York, USA: ACM Press; 1968, p. 517–24. 10.1145/800186.810616.

[16] Dormann, C. F. et al. Collinearity: a review of methods to deal with it and a simulation study evaluating their performance. *Ecography* **36**, 27–46. 10.1111/J.1600-0587.2012.07348.X (2013).

[17] Naimi, B. & Araújo, M. B. sdm: a reproducible and extensible R platform for species distribution modelling. *Ecography* **39**, 368–375. 10.1111/ecog.01881 (2016).

[18] Araújo, M. B. & Guisan, A. Five (or so) challenges for species distribution modelling. *J. Biogeogr.* **33**, 1677–1688. 10.1111/j.1365-2699.2006.01584.x (2006).

[19] Allouche, O., Tsoar, A. & Kadmon, R. Assessing the accuracy of species distribution models: Prevalence, kappa and the true skill statistic (TSS). *J. Appl. Ecol.* **43**, 1223–1232. 10.1111/J.1365-2664.2006.01214.X (2006).

[20] Burnham KP, Anderson DR. Model Selection and Multimodel Inference. 1998.

[21] Guisan, A. & Thuiller, W. Predicting species distribution: Offering more than simple habitat models. *Ecol. Lett.* **8**, 993–1009. 10.1111/j.1461-0248.2005.00792.x (2005). 34517687 10.1111/j.1461-0248.2005.00792.x

[22] Breiman, L. Random forests. *Mach. Learn.* **45**, 5–32 (2001).

[23] Hastie T, Tibshirani R. Generalized Additive Models. Monographs on Statistics & Applied Probability Chapman and Hall/CRC 1990;1.

[24] Phillips, S. J., Anderson, R. P. & Schapire, R. E. Maximum entropy modeling of species geographic distributions. *Ecol. Modell.* **190**, 231–259. 10.1016/j.ecolmodel.2005.03.026 (2006).

[25] McCullagh, P. *Generalized linear models* (Routledge, 2018).

[26] Araújo, M. B. & New, M. Ensemble forecasting of species distributions. *Trends Ecol. Evol.* **22**, 42–47. 10.1016/j.tree.2006.09.010 (2007). 17011070 10.1016/j.tree.2006.09.010

[27] Araújo, M. B. & Williams, P. H. Selecting areas for species persistence using occurrence data. *Biol. Conserv.* **96**, 331–345. 10.1016/S0006-3207(00)00074-4 (2000).

[28] Elith, J., Kearney, M. & Phillips, S. The art of modelling range-shifting species. *Methods Ecol. Evol.* **1**, 330–342. 10.1111/j.2041-210x.2010.00036.x (2010).

[29] Barbosa, A. M., Real, R. & Mario, V. J. Transferability of environmental favourability models in geographic space: The case of the Iberian desman (*Galemys pyrenaicus*) in Portugal and Spain. *Ecol Modell* **220**, 747–754. 10.1016/J.ECOLMODEL.2008.12.004 (2009).

[30] Palomo, L. J. & Gisbert, J. y Blanco JC. Atlas y Libro Rojo de los Mamíferos Terrestres de España. Dirección General Para La Biodiversidad-SECEM-SECEMU, 588 (Madrid, 2007).

[31] Schoener, T. W. The Anolis Lizards of Bimini: Resource Partitioning in a Complex Fauna. *Ecology* **49**, 704–726. 10.2307/1935534 (1968).

[32] R Core Team. R: A language and environment for statistical computing. R Foundation for Statistical Computing (Vienna, Austria, 2024). https://www.R-project.org/.

[33] Mysterud, A., Madslien, K., Herland, A., Viljugrein, H. & Ytrehus, B. Phenology of deer ked (*Lipoptena cervi*) host-seeking flight activity and its relationship with prevailing autumn weather. *Parasit Vectors* **9**, 1–6. 10.1186/S13071-016-1387-7/FIGURES/2 (2016). 26897626 10.1186/s13071-016-1387-7 PMC4761182

[34] Hackman, W., Rantanen, T. & Vuojolahti, P. Immigration of *Lipoptena cervi* (Diptera, Hippoboscidae) in Finland, with notes on its biology and medical significance. *Notulae Entomologicae* **63**, 7 (1983).

[35] Tiawsirisup, S. et al. Possible role of *Lipoptena fortisetosa* (Diptera: Hippoboscidae) as a potential vector for *Theileria* spp. in captive Eld’s deer in Khao Kheow open zoo, Thailand. *Acta Trop.* **237**, 106737. 10.1016/j.actatropica.2022.106737 (2023). 36341781 10.1016/j.actatropica.2022.106737

[36] Meier, C. M., Bonte, D., Kaitala, A. & Ovaskainen, O. Invasion rate of deer ked depends on spatiotemporal variation in host density. *Bull Entomol Res* **104**, 314–322. 10.1017/S0007485314000042 (2014). 24521661 10.1017/S0007485314000042

[37] Kurina, O., Kirik, H., Õunap, H. & Õunap, E. The northernmost record of a blood-sucking ectoparasite, *Lipoptena fortisetosa* Maa (Diptera: Hippoboscidae), in Estonia. *Biodivers. Data J.* **7**, e47857. 10.3897/BDJ.7.E47857 (2019). 31875091 10.3897/BDJ.7.e47857 PMC6925061

[38] Kynkäänniemi, S. M., Kortet, R. & Laaksonen, S. Range expansion and reproduction of the ectoparasitic deer ked (*Lipoptena cervi*) in its novel host, the Arctic reindeer (*Rangifer tarandus tarandus*), in Finland. *Parasitol Res* **119**, 3113–3117. 10.1007/S00436-020-06817-X/TABLES/2 (2020). 32699937 10.1007/s00436-020-06817-x PMC7431400

[39] Gałęcki, R., Jaroszewski, J., Xuan, X. & Bakuła, T. Temporal-microclimatic factors affect the phenology of *Lipoptena fortisetosa* in central European forests. *Animals* **10**, 1–12. 10.3390/ani10112012 (2020). 10.3390/ani10112012 PMC7692670 33139594

[40] Yatsuk, A., Triseleva, T., Matyukhin, A., Safonkin, A. & Nartshuk, E. Two cases of introducing *Lipoptena fortisetosa* Maa (Diptera: Hippoboscidae) into Europe through different deer species. *J. Nat. Hist.* **58**, 1787–1801. 10.1080/00222933.2024.2395905 (2024).

[41] Usai, F., Dini, F. M., Guarniero, I., Bellinello, E. & Stancampiano, L. The enigmatic case of *Lipoptena* sp. in the Bosco della Mesola Nature Reserve (Italy). *Med Vet Entomol.*10.1111/MVE.70002 (2025). 40831427 10.1111/mve.70002 PMC12865754

[42] Oboňa, J. et al. Is *Ornithoctona laticornis* (Diptera: Hippoboscidae) expanding its range from Africa into Europe? First confirmed record in Romania. *Int. J. Parasitol. Parasites Wildl* **27**, 101089. 10.1016/J.IJPPAW.2025.101089 (2025). 40520771 10.1016/j.ijppaw.2025.101089 PMC12166706

[43] Maślanko, W., Bartosik, K., Raszewska-Famielec, M., Szwaj, E. & Asman, M. Exposure of Humans to Attacks by Deer Keds and Consequences of Their Bites-A Case Report with Environmental Background. *Insects* **11**, 1–9. 10.3390/INSECTS11120859 (2020). 10.3390/insects11120859 PMC7761680 33287132

[44] Rantanen, T., Reunala, T., Vuojolahti, P. & Hackman, W. Persistent pruritic papules from deer ked bites. *Acta Derm Venereol* **62**, 307–311. 10.2340/0001555562307311 (1982). 6183862

[45] Laukkanen, A., Ruoppi, P. & Mäkinen-Kiljunen, S. Deer ked-induced occupational allergic rhinoconjunctivitis. *Ann Allergy Asthma Immunol* **94**, 604–608. 10.1016/S1081-1206(10)61141-6 (2005). 15945565 10.1016/S1081-1206(10)61141-6

[46] Decastello, A. & Farkas, R. Anaphylactic reaction following forest fly (*Hippobosca equina)* bite: A human case. *Clin. Exp. Med. J.* **4**, 193–198. 10.1556/CEMED.4.2010.1.19 (2010).

