# Supplementary material for: Ecology and distribution of the recently discovered ked Lipoptena andaluciensis (Diptera: Hippoboscidae) in Spain
Source: Sci Rep. 2025 Nov 28;15:42728. doi: 10.1038/s41598-025-26742-2 (PMC12663212; doi:10.1038/s41598-025-26742-2)
Supplement: Supplementary file 1 — Supplementary Material 1 [file 41598_2025_26742_MOESM1_ESM.docx]

**Supplementary material**

**Table S1.** Final environmental variables selected for spatio-ecological modeling, displaying Variance Inflation Factor (VIF) scores. Land cover percentages were calculated within 2-kilometer grid cells; climatic and topographic variables were sourced from global databases. VIF analysis conducted using the *terra* package in R confirmed no critical multicollinearity concerns (all values <5)

| Variable Used in the model | VIF |  |
| --- | --- | --- |
| Shrubs | *1.62* | Percentage of shrublands in 2km grid cell |
| Bio.2 | *4.7* | Mean Diurnal Range |
| Forest | *4.3* | Percentage of Forest in 2km grid cell |
| DEM | *3.06* | Digital Elevation model |
| Soil | *1.89* | Soil Temperature |
| LSTMean | *1.49* | Land Surface Temperature |
| Crop | *4.09* | Percentage of croplands in 2km grid cell |
| NDVIMean | *3.65* | Normalized Difference Vegetation Index |

**
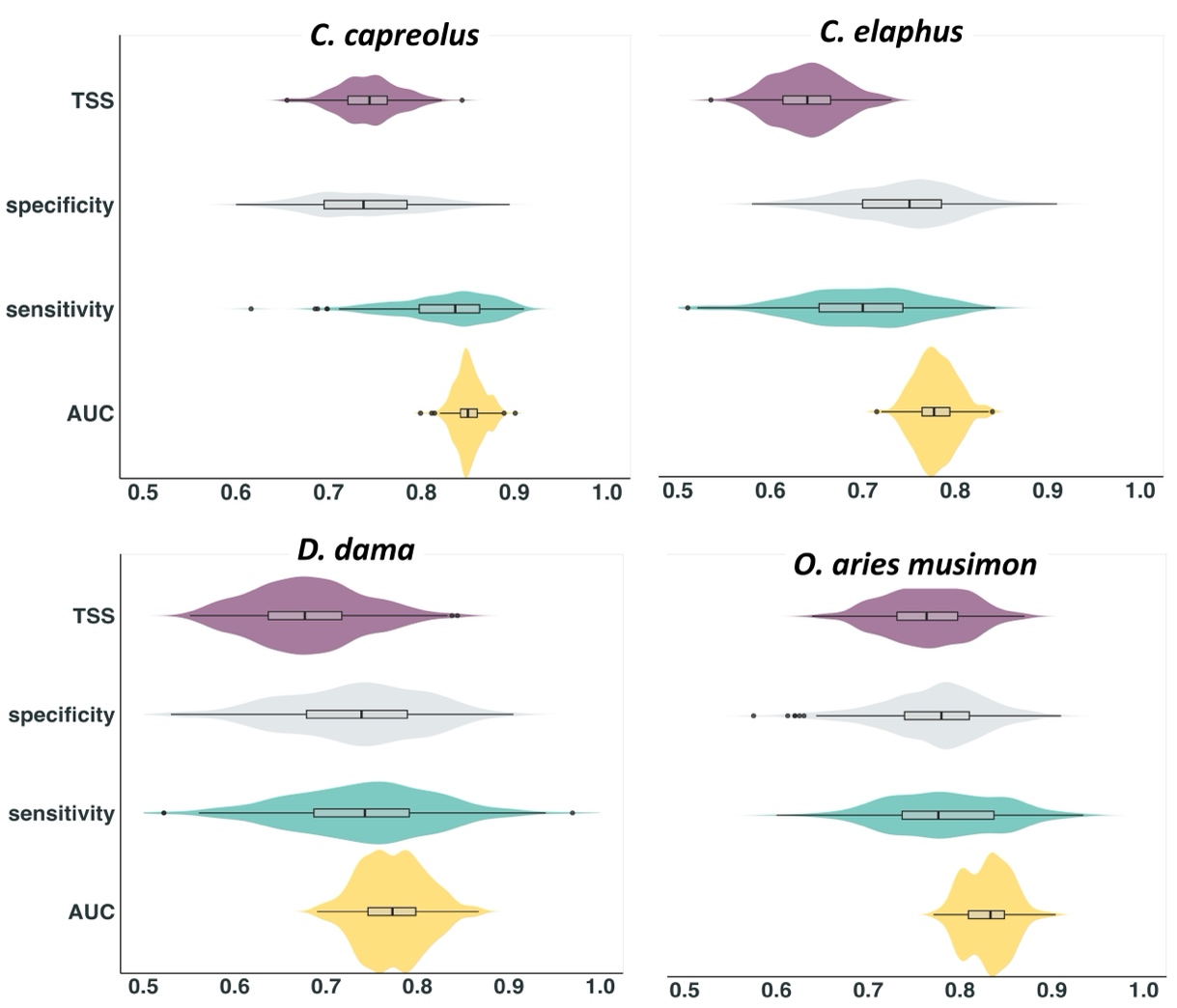
**

**Figure S1.** Violin plots show the mean model performance for four mammalian species using AUC (Area Under the ROC Curve), TSS (True Skill Statistic), Sensitivity (true positive rate), and Specificity (true negative rate) based on an ensemble of four modeling techniques. Performance was evaluated using a test dataset generated through five-fold cross-validation and bootstrapping partitioning. An AUC value >0.80 indicates very good performance; 0.7 < AUC ≤ 0.8 indicates good performance; 0.5 < AUC ≤ 0.7 is considered acceptable; and AUC ≤ 0.5 reflects poor performance or random prediction. For TSS, values >0.75 indicate very good performance; 0.5 < TSS ≤ 0.75 indicates good performance; 0.2 < TSS ≤ 0.5 reflects low performance; and TSS ≤ 0.2 indicates poor or random prediction.

**Figure S2.** Multivariate Environmental Similarity Surface (MESS) map showing how similar the environmental conditions across the study area are to those used to train the species distribution model. Areas in white, particularly in northern Spain, represent high environmental dissimilarity, meaning conditions there differ substantially from the model’s calibration data. These regions may involve extrapolation beyond the species’ known environmental range, so predictions in these areas should be interpreted with caution.

**Figure S3.** Environmental niche similarity between L. andaluciensis and its four potential mammalian hosts based on principal component analysis (PCA) of environmental variables. The x-axis (PC1) and y-axis (PC2) represent the first two principal components, summarizing the major environmental gradients across the study area. Green areas indicate high environmental similarity (niche overlap) between the parasite and the host species, while red areas reflect lower similarity.
